# CpG Island Methylation in Human Lymphocytes Is Highly Correlated with DNA Sequence, Repeats, and Predicted DNA Structure 

**DOI:** 10.1371/journal.pgen.0020026

**Published:** 2006-03-03

**Authors:** Christoph Bock, Martina Paulsen, Sascha Tierling, Thomas Mikeska, Thomas Lengauer, Jörn Walter

**Affiliations:** 1 Max-Planck-Institut für Informatik, Saarbrücken, Germany; 2 Universität des Saarlandes, Genetik/Epigenetik, Saarbrücken, Germany; The Babraham Institute, United Kingdom

## Abstract

CpG island methylation plays an important role in epigenetic gene control during mammalian development and is frequently altered in disease situations such as cancer. The majority of CpG islands is normally unmethylated, but a sizeable fraction is prone to become methylated in various cell types and pathological situations. The goal of this study is to show that a computational epigenetics approach can discriminate between CpG islands that are prone to methylation from those that remain unmethylated. We develop a bioinformatics scoring and prediction method on the basis of a set of 1,184 DNA attributes, which refer to sequence, repeats, predicted structure, CpG islands, genes, predicted binding sites, conservation, and single nucleotide polymorphisms. These attributes are scored on 132 CpG islands across the entire human Chromosome 21, whose methylation status was previously established for normal human lymphocytes. Our results show that three groups of DNA attributes, namely certain sequence patterns, specific DNA repeats, and a particular DNA structure, are each highly correlated with CpG island methylation (correlation coefficients of 0.64, 0.66, and 0.49, respectively). We predicted, and subsequently experimentally examined 12 CpG islands from human Chromosome 21 with unknown methylation patterns and found more than 90% of our predictions to be correct. In addition, we applied our prediction method to analyzing Human Epigenome Project methylation data on human Chromosome 6 and again observed high prediction accuracy. In summary, our results suggest that DNA composition of CpG islands (sequence, repeats, and structure) plays a significant role in predisposing CpG islands for DNA methylation. This finding may have a strong impact on our understanding of changes in CpG island methylation in development and disease.

## Synopsis

DNA methylation is the only epigenetic mechanism in eukaryotes that is known to directly modify the DNA. It plays an important role for gene control during development and cell differentiation, and it is a promising therapeutic target in cancer research. While a genome-wide picture of DNA methylation patterns is currently emerging, we have only fragmentary knowledge about the linkage between DNA methylation and other genomic attributes such as DNA sequence and structure, repetitive elements, or sequence conservation. The authors fill this gap by reporting on a comprehensive bioinformatical analysis of DNA methylation on human Chromosome 21—and in part, extending to other regions of the human genome. They report new associations that will help elucidate the functions of DNA methylation along the human genome. Furthermore, the authors show that their findings can be applied to predicting DNA methylation patterns from genome sequence. Such predictions have the potential of speeding up genome-wide epigenetic profiling: It may be possible to focus experimental resources on a few selected areas while bioinformatics procedures are applied to the bulk of the genome.

## Introduction

DNA methylation is a frequent biochemical modification of eukaryotic DNA [1–6]. In humans, it affects the C5 position of cytosines that belong to CpG dinucleotides (i.e., a cytosine directly followed by a guanine).

CpG dinucleotides are distributed unevenly across the human genome. In non-coding DNA, CpG dinucleotides are 4-fold underrepresented compared to the frequency of the other dinucleotides [7,8], with the remarkable exception of so-called CpG islands. There, CpGs are approximately as frequent as one would expect from single base pair frequencies. For practical reasons, CpG islands are usually defined as sequence stretches that fulfill three conditions [9]: (i) GC content above 50%, (ii) ratio of observed versus expected number of CpG dinucleotides above 0.6, and (iii) more than *n* base pairs in length (we use *n* = 400 in accordance with the source of our dataset [10]). CpG islands rarely exceed 5,000 base pairs and are often associated with functional elements. In particular, CpG islands overlap with the promoter regions of 50% to 60% of human genes, including most housekeeping genes [11,12].

As DNA methylation in the human genome is largely confined to CpG dinucleotides, it is not surprising that the distribution of DNA methylation along the genome is closely intertwined with CpG frequencies. The classical view is that almost all dispersed CpG dinucleotides in the human genome are methylated by default, whereas CpG dinucleotides inside CpG island promoters are typically unmethylated in normal (i.e., non-neoplasic, non-senescent) tissue [1]. However, exceptions have been known for a long time, such as de novo methylation during cell differentiation [13], imprinting [3], and X-chromosome inactivation [14]. Strong biallelic DNA methylation of CpG island promoters is associated with stable silencing of neighboring or associated genes and constitutes a frequent event in cancer progression [15].

Initial chromosome-wide and genome-wide studies of CpG island methylation indicate that a sizeable fraction of CpG islands is methylated in normal tissue [10,16]. However, little is known about the mechanisms that lead to methylation of certain CpG islands while leaving others unmethylated, and it is unclear whether these two groups can be identified by characteristic attributes. Inspired by recent exploratory results pointing towards a significant role of local DNA sequences in predetermining DNA methylation at the nucleotide level (i.e., CpG dinucleotides instead of CpG islands) [17,18], as well as for aberrant methylation [19], we performed a comprehensive analysis of the association of DNA-related features and normal CpG island methylation on human Chromosome 21. Our results show that DNA sequence patterns, repeat frequencies, and predicted DNA structure are highly correlated with CpG island methylation. We successfully used this association to predict the methylation status for new CpG islands.

## Results

In this study, we explore the relationship between DNA methylation and various DNA-related features at the biologically functional CpG island level, using computational epigenetics methodology. Based on a dataset published by Yamada et al., comprising all CpG islands on the non-repetitive parts of human Chromosome 21 [10] and a compiled list of 1,184 DNA-related attributes, we quantify the correlation between CpG island methylation and eight attribute classes: (1) DNA sequence properties and patterns, (2) repeat frequency and distribution, (3) CpG island frequency and distribution, (4) predicted DNA structure, (5) gene and exon distribution, (6) predicted transcription factor binding sites, (7) evolutionary conservation, and (8) single nucleotide polymorphisms (SNPs). We identify the attributes that are most predictive in distinguishing between methylated and unmethylated CpG islands and we show that it is possible to predict CpG island methylation from DNA-related features with high accuracy. Finally, we validate our results both experimentally on Chromosome 21 and bioinformatically on data from the Human Epigenome Project (HEP) [20].

### Identification of DNA-Related Attributes That Distinguish Methylated CpG Islands from Their Unmethylated Counterparts

As a first step towards understanding the relationship between DNA-related attributes and CpG island methylation, we statistically compared the distributions between methylated and unmethylated CpG islands for all attributes in our list (see Dataset S1 for the full list of *p*-values and Materials and Methods for an overview of attribute definitions). Using a conservative significance threshold, 41 attributes showed significant differences (Table 1).

**Table 1:**
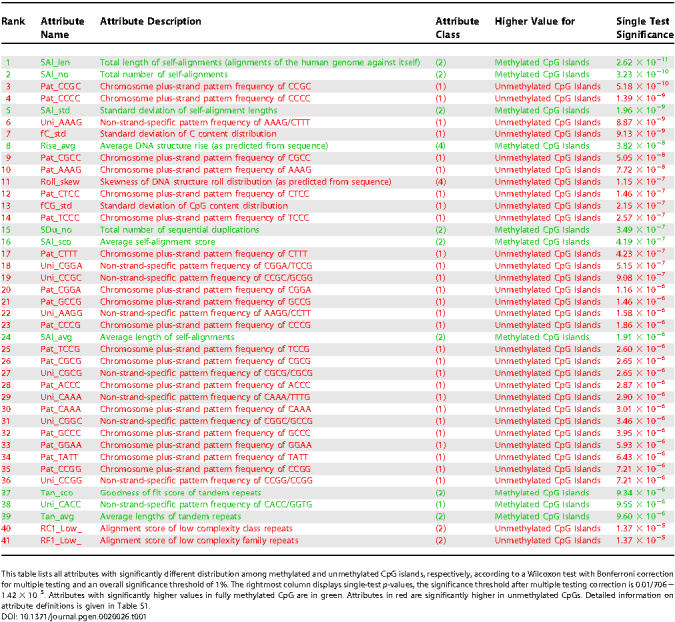
DNA-Related Attributes Differ Significantly between Methylated and Unmethylated CpG Islands

Of the significant attributes, the majority are frequencies of GC-rich and CpG-rich DNA sequence patterns, which are over-represented in unmethylated CpG islands. Non-strand-specific patterns and patterns that are strand-specific relative to the chromosomal plus-strand occur with similar frequency and composition. Several attributes that refer to repetitive DNA are more frequent in methylated CpG islands (such as segmental duplications, self chain alignments, and tandem repeats).

**Figure 1:**
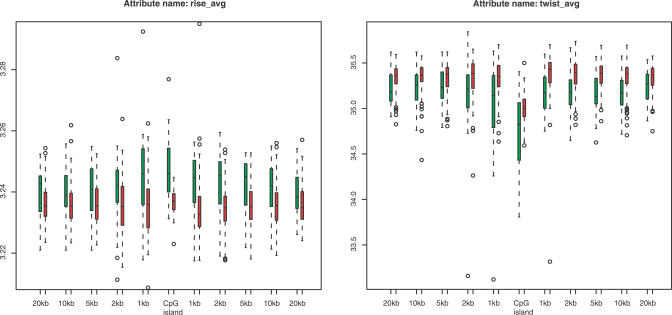
Predicted DNA Structure Differs in the Neighborhood of Methylated CpG Islands Compared with Their Unmethylated Counterparts The diagram on the left shows boxplots of the predicted DNA rise distribution over the CpG island and the ten sequence windows from −20 kb to 20 kb surrounding the CpG island (averaged over all 132 CpG islands in the Chromosome 21 dataset). Green bars (left) correspond to methylated CpG islands, red bars (right) to unmethylated CpG islands. The diagram on the right shows similar information for the predicted DNA twist.

Interestingly, two aspects of predicted DNA structure, most prominently the average rise of the DNA helix, also show different distributions for methylated and unmethylated CpG islands (see Olson et al*.* [21] for an overview of DNA structure nomenclature). The role of predicted DNA structure becomes even more pronounced when considering not only the CpG island itself, but also the −20-kilobase (kb) to +20-kb sequence windows surrounding it. In that case, the predicted average rise and the predicted average twist are the second and third most significant among all attributes (Dataset S1, second worksheet). An inspection of the corresponding boxplots (Figure 1) shows that the predicted DNA rise increases on average within CpG islands compared to the genomic neighborhood, whereas the twist decreases. However, this effect is much stronger for methylated than for unmethylated CpG islands. Hence, methylated CpG islands tend to co-locate with areas of unusual predicted DNA structure.

Furthermore, it is apparent from Table 1 that a single pattern is over-represented in methylated CpG islands, namely the non-strand-specific CACC/GGTG pattern. Because this pattern contains a TpG, in contrast to the CpG-rich patterns that are frequent in unmethylated CpG islands, it is tempting to argue that this pattern may be the result of sporadic deamination of original GG^M^CG patterns (such mutations are less likely to be repaired for methylated CpGs [6]). In order to test whether differential CpG → TpG mutation rates may be a source of differential pattern frequencies between methylated and unmethylated CpG islands, we compared the palindromic pattern CGCG with the non-strand-specific pattern TGTG/CACA, which can evolve from the former pattern by two subsequent deamination mutations.

In agreement with our hypothesis, we find the CGCG pattern more frequently in unmethylated CpG islands (mean of 12.61 occurrences per kb) compared to methylated CpG islands (7.15 occurrences per kb) and the TGTG/CACA pattern more frequently in methylated CpG islands (10.92 occurrences per kb) compared to unmethylated CpG islands (2.93 occurrences per kb). In both cases, *p*-values were below 0.001 according to a Wilcoxon test. These results suggest that during evolution, higher rates of germline CpG → TpG mutation occurred in those CpG islands that are methylated in human lymphocytes compared to those that are unmethylated.

Finally, we analyzed the dataset for evidence of experimental bias. Because restriction enzyme digestion was used to discriminate between methylated and unmethylated CpG islands [10], incomplete digestion is a potential error source. In this case, we would expect the HpaII recognition site (CCGG) to behave significantly different from patterns that are never cut. However, we observe no indication of this in our attribute statistics (Dataset S1, first worksheet). Five out of ten GC-rich and CpG-containing sequence patterns have higher *p*-values than the CCGG pattern (CCGC, CGCC, GCCG, CCCG, and CGCG), while the same number of patterns has a lower *p*-value (GCGC, CGGC, GCGG, CGGG, and GGCG). We conclude that the experimental method that was applied by Yamada et al*.* is sufficiently unbiased for our analysis.

### Quantification of the Association between DNA-Related Attributes and CpG Island Methylation

Strikingly, all attributes that were significantly different between methylated and unmethylated CpG islands (Table 1) fall into three (out of eight) attribute classes: (1) DNA sequence properties and patterns, (2) repeat frequency and distribution, and (4) predicted DNA structure. In order to investigate this observation more systematically, we calculated the group-wise correlation between CpG island methylation and each of the eight attribute classes.

In contrast to single-attribute correlation coefficients, group-wise correlations are able to capture combined effects of interacting attributes (e.g., when neither A nor B has any significant impact on methylation alone, but the combined presence of both is highly associated with a certain methylation status). Support vector machines (SVMs) have been successfully employed to detect such combined effects. Therefore, we trained a (linear) SVM to predict CpG island methylation and tested its performance on unseen data (10-fold cross-validation). Then, we calculated the correlation coefficient between the SVM's predictions on unseen data and the correct values, averaging over 20 independent cross-validation runs. This measure gives us a conservative estimate for the group-wise correlation between the attribute group and CpG island methylation—conservative because it may well be that the SVM does not capture all information on CpG island methylation that is present in the attribute group, while it is highly unlikely to predict methylation correctly over multiple runs if not enough information is contained in the attributes.

Our results substantiate the observation that three classes of DNA-related attributes are distinctly associated with CpG island methylation status (Table 2, experiments 1 to 8): (1) DNA sequence properties and patterns, as well as (2) repeat frequency and distribution are correlated with CpG island methylation at medium to high rates (correlation coefficient of 0.635 and 0.657, respectively); whereas (4) predicted DNA structure falls behind (0.486). Three of the remaining attribute classes exhibit weak correlation with CpG island methylation, namely (5) gene and exon distribution (0.300), (8) SNPs (0.286), and (3) CpG island frequency and distribution (0.045). (6) Predicted transcription factor binding sites and (7) evolutionary conservation are uncorrelated with CpG island methylation (−0.021 and 0.000, respectively). Furthermore, the combination of all eight attribute classes results in a higher correlation value than any single class (0.740), indicating that at least some attribute classes capture complementary information.

**Table 2:**
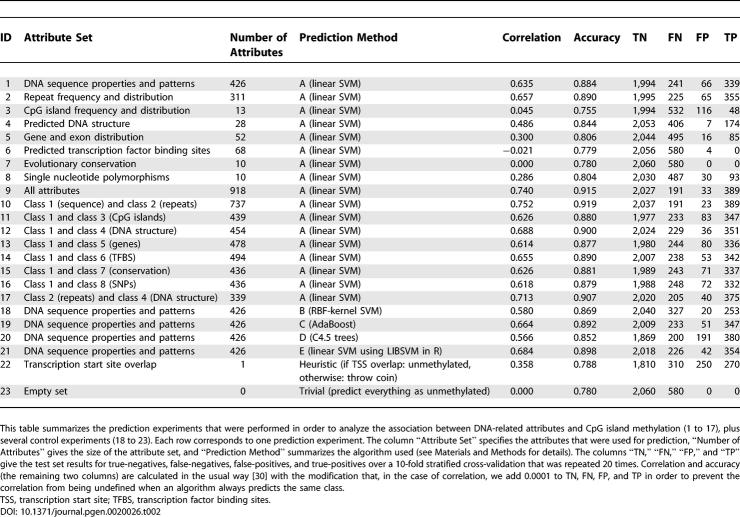
The Predictive Power of Attribute Classes Differs Remarkably; Control Experiments Confirm the Appropriateness of the Prediction Method

To quantify the degree of complementarity and to find out which attribute classes are positively correlated with DNA methylation only due to indirect or secondary effects, we applied the following strategy. Given two attribute classes, we calculate the correlation for both classes separately and for the combination of both. If the latter is higher than any of the former, we can conclude that the attributes complement each other. Comparing DNA sequence with all other attribute classes reveals that only (2) repeat frequency and distribution and (4) predicted DNA structure give rise to an increased correlation when combined with (1) DNA sequence properties and patterns, by 18.4% and 8.3%, respectively (Table 2, experiments 10 to 16). However, among these three classes, all combinations significantly increase the correlation (Table 2, experiments 10, 12, and 17).

Therefore, we conclude that three attribute classes, namely (1) DNA sequence properties and patterns, (2) repeat frequency and distribution, and (4) predicted DNA structure are correlated with CpG island methylation on their own right (primary effect). The remaining attribute classes are either not correlated with CpG island methylation at all (class 7 evolutionary conservation and class 8 predicted transcription factor binding sites), or their correlations are secondary, explainable by their co-location with certain DNA sequence patterns alone (class 3 CpG island frequency and distribution, class 5 gene and exon distribution, and class 8 SNPs).

### Prediction of CpG Island Methylation Status from DNA-Related Attributes

While the previous section was concerned with quantifying the relative contribution of different attribute classes to explaining CpG island methylation, the same methodology can be used to predict the methylation status of new CpG islands. Here we report the prediction performance of our method and we address potential limitations.

Without prior knowledge it is sensible to include all 918 non-zero attributes simultaneously in order to achieve best prediction results. In a 10-fold stratified cross-validation of a linear SVM, which we repeated 20 times with different random partitions, this setup resulted in an average correlation of 0.74, a test set accuracy of 91.5%, a specificity of 98.4%, and a sensitivity of 67.1% (Table 2, experiment 9).

In order to test the appropriateness of the prediction method that we used (SVM with linear kernel), we performed several control experiments employing other state-of-the-art machine learning algorithms [22], namely SVM with radial basis function kernel, AdaBoost using tree stumps, C4.5 tree generator, and a second widely used implementation of a linear SVM. The results show that performances of all methods lie within the same range (Table 2, experiments 18 to 21).

Next, we investigated how prediction accuracies vary between CpG islands that are located at different positions relative to their closest annotated gene. For this analysis, we regard a single CpG island as reliably predicted if its prediction is correct in at least 15 out of 20 randomized cross-validations, and we manually assigned each of the 132 CpG islands of the Chromosome 21 dataset to one of the following categories (Dataset S2):

Category 1: Promoter CpG islands, defined as overlapping with the transcription start site of an annotated gene: 80 cases fall into this category, of which 78 are unmethylated.

Category 2: Intragenic CpG islands, defined as overlapping introns and/or exons of an annotated gene, but not the transcription start site: 24 cases fall into this category, of which 12 are unmethylated.

Category 3: Gene-terminal CpG islands, defined as overlapping mainly the last exon and/or the 3′ UTR of an annotated gene: six cases fall into this category, of which one is unmethylated.

Category 4: Intergenic CpG islands, defined as not showing any overlap with an annotated human gene: 22 cases fall into this category, of which 12 are unmethylated.

Our results show that prediction accuracy is highest for promoter CpG islands, where 77 unmethylated cases and one methylated case are predicted correctly in more than 15 out of 20 runs (98% accuracy); the second methylated case is predicted correctly in seven out of 20 runs (Ensembl gene *ENSG00000197597*) and the one remaining unmethylated case is correctly predicted in only three runs (Ensembl gene *ENSG00000160207*). In categories 2, 3, and 4, the number of methylated and unmethylated CpG islands is almost balanced, thus prediction is much more difficult. Nevertheless, prediction accuracies stay high: For intragenic CpG islands, 20 cases are predicted correctly in more than 15 runs (83% accuracy). Among the gene-terminal CpG islands, four cases are predicted correctly in more than 15 runs (67% accuracy), and of all intergenic CpG islands, 18 are correctly predicted in more than 15 runs (82% accuracy).

In conclusion, our method achieves high prediction accuracy for CpG islands from all four categories. Finally, we note that the method significantly outperforms a heuristic prediction which relies on transcriptional start site overlap alone (Table 2, experiment 22), and that the very high specificity of the method (98.4%) facilitates chromosome-wide screening for methylated CpG islands, giving rise to a low number of false-positives.

### Experimental Validation by Bisulphite Sequencing

In order to further substantiate the reliability of our method, we experimentally validated its predictions for 12 CpG islands. To that end, we first predicted the methylation state of all CpG islands from Chromosome 21 that were not part of the original dataset [10]; either because they did not match the strict CpG island criteria imposed by Yamada et al*.* or because they (marginally) overlap with repetitive DNA. Next, we selected eight CpG islands that were predicted as unmethylated and four CpG islands that were predicted as methylated, and we experimentally determined their methylation status in human peripheral blood by bisulphite sequencing.

Hence, while keeping species (human) and chromosome (21) identical, we varied experimental technique (bisulphite sequencing instead of restriction enzyme digestion), cell type (peripheral blood instead of lymphocytes), sample origin (healthy European female instead of healthy unspecified), and—of course—the CpG island. In the selection of validation CpG islands, we did not stratify for CpG island categories (see previous section) because we wanted to assess the method's overall performance across all categories of CpG islands.

The experimental results (Table 3) show that our prediction was correct in ten out of 11 cases (*p*-value below 0.01). The 12th case, predicted as methylated, showed an incomplete yet significant methylation of 54%. Hence, our method can predict CpG island methylation with high accuracy on a previously unknown test set.

**Table 3:**
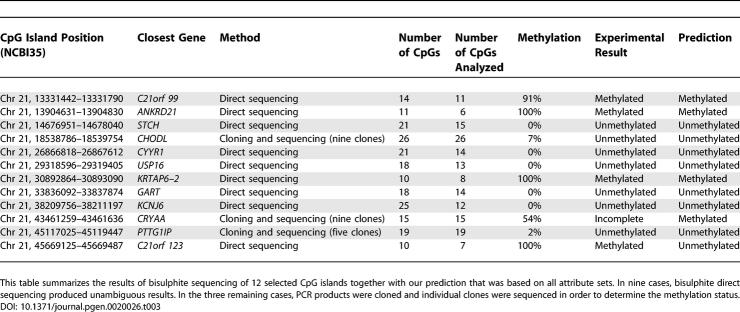
Twelve CpG Islands Were Analyzed Experimentally to Validate Our Predictions

### Comparison with HEP Dataset

The DNA methylation data from the HEP pilot study [20] gives us the opportunity to assess the generality of our method and the transferability of the predictions that we obtain from the Chromosome 21 dataset. A priori, one would not expect a high degree of transferability because the HEP data vary from the Chromosome 21 data that were used to develop the method in several important aspects. First, almost 90% of amplicons for which DNA methylation profiles were established do not fulfill CpG island properties. Second, the HEP did not analyze lymphocytes but a variety of other tissues (adipose, brain, breast, liver, lung, muscle, and prostate). Third, all analyzed sequences belong to the relatively small and exceptional major histocompatibility complex on Chromosome 6.

In order to make the HEP dataset accessible to our method, which works on CpG islands (or DNA stretches of comparable length), we calculated the average CpG dinucleotide methylation for every HEP amplicon, and we defined a threshold to distinguish methylated from unmethylated amplicons (see Materials and Methods for details). Next, we trained our method on the Chromosome 21 dataset and predicted the methylation status of all HEP amplicons, in a similar way as was done for the experimental validation in the previous section. The results show a prediction accuracy that is low but still better than random (correlation = 0.15, accuracy = 74.7%, true-negatives = 10, false-negatives = 16, false-positives = 37, true-positives = 147). Hence, there seems to be a core association between DNA-related features and CpG island methylation that is similar or identical across tissues and genomic locations. This association can be specified further by a comparison of prediction error rates. First, we observe a remarkably low false-negative rate of 10%. In other words, the characteristics that were learned to predict CpG islands as methylated in lymphocytes are to some extent transferable across tissues and genomic locations, giving rise to a low false-negative rate on the HEP dataset. Second, the false-positive rate was 8-fold higher than the corresponding false-negative rate (79%), indicating that it is difficult to transfer the DNA-related characteristics of unmethylated cases between the two datasets.

Next, we analyzed to what degree the prediction performance improves when the method is provided with a more adequate training dataset, i.e., when it is permitted to learn the characteristics that are unique to the HEP dataset. To that end, we trained and evaluated our prediction method in a cross-validation on the HEP dataset using all eight attribute classes. Taking into account all HEP amplicons, this resulted in a sharp increase in prediction performance, with a correlation of 0.47 and an accuracy of 82.4% (true-negatives = 25.7, false-negatives = 15.6, false-positives = 21.3, true-positives = 147.4, averaged over 20 independent cross-validation runs). A further performance increase was observed when we repeated the analysis on amplicons that do not deviate too strongly from the CpG island characteristic, for which the prediction method was developed. We sorted all amplicons by the ratio of observed versus expected CpG dinucleotide frequency, and ran a separate training and prediction analysis for the top, middle, and bottom 70 cases. Results show a correlation of 0.59 for the top group and 0.73 for the middle group (one third of the amplicons in the top group and none in the middle group fulfill CpG island properties). In contrast, predictions fail for the bottom group (correlation = −0.02) where unmethylated cases are rare (six out of 70), possibly because sample size is too small or because these cases behave more randomly.

These results indicate that our prediction method is also well-suited to predict the average methylation status for sequences that are not necessarily CpG islands, at least when a suitable training set is provided and CpG dinucleotide frequency is not too low.

Finally, because the HEP dataset contains methylation information for seven different tissues it should be possible, in principle, to detect evidence of tissue-specific methylation regulation, for example, binding site patterns of tissue-specific transcription factors. Therefore, one would expect that the prediction performance of our method was higher if trained on data from only one tissue, compared to the combination of all tissues, at least when focusing only on the most tissue-specific amplicons. However, we find no evidence for this in our dataset. Instead, prediction performances for individual tissues closely resemble the average case (unpublished data). There are several possible explanations for the method's failure to learn tissue-specific methylation information from the HEP dataset. On the one hand, tissue-specific methylation may be largely uncorrelated with the sequence-related attributes that we analyzed. On the other hand, the dataset may simply be too small. In fact, only between five and 19 out of 210 amplicons per tissue deviate from the “default” state calculated as the consensus methylation over all tissues.

## Discussion

We have shown that CpG island methylation can be predicted from DNA sequence and that we may be able to enhance our understanding of the biology that controls methylation in vivo by predictive bioinformatics analysis. First, we identified DNA-related attributes that discriminate strongly between methylated and unmethylated CpG islands in human lymphocytes. Second, we quantified the correlation of CpG island methylation with eight groups of DNA-related attributes and found DNA sequence patterns, repeat frequencies, and predicted DNA structure to be the key contributors. Third, we developed a machine-learning method that can predict the methylation status of unknown CpG islands and we validated the accuracy and reliability of this method both statistically and experimentally.

Our results raise a number of questions concerning our current view of CpG island methylation. While it is apparent from the attribute statistics (Table 1) that CpG-rich patterns are over-represented in unmethylated CpG islands, we found no evidence of a simple yet accurate relationship between CpG island methylation on the one hand and CpG dinucleotide frequency, observed versus expected ratio, or the density of restriction sites such as CCGG (HpaII) and CGCG (HhaI), on the other hand. Instead, methylated and unmethylated CpG islands each seem to be characterized by a relatively complex combination of the presence or absence of certain sequence motifs and attributes of DNA structure. However, we did not find any evidence of a combinatorial “DNA sequence code” for methylation; hence, we suggest that the individual sequence and structure attributes contribute to the preferred methylation state of a CpG island independently and possibly in an additive way.

For repetitive DNA, the situation is more straightforward: CpG islands that significantly overlap with a tandem repeat or a segmental duplication are methylated in almost all cases, which is in line with the long-known fact that tandem repeats form heterochromatin [23]. Unfortunately, we could not address the influence of retrotransposons such as SINE and LINE elements on CpG island methylation since our dataset [10] only contains CpG islands that are not suppressed by RepeatMasker [24].

Based on these observations, we propose that each CpG island can be assigned a degree of methylation propensity that is encoded in its DNA. This default state is what our prediction method captures. For a relatively small number of CpG islands the default state is overruled by biological processes such as tissue differentiation, X-chromosome inactivation, or imprinting, which enforce a certain methylation state. In normal tissue and on the autosomes, these effects seem to affect only a minority of CpG islands—otherwise the high prediction accuracies that we observe could not be argued. This is consistent with the observation that only around 5% of CpG islands are differentially methylated in a tissue-specific fashion [25], and that the effect of imprinting is even more limited. However, since our prediction analysis was constrained to data from two chromosomes and few tissues, such a far-reaching interpretation of our results has to be taken with care. It will be interesting to see whether CpG islands that consistently deviate from their default methylation state due to monoallelic methylation (imprinting, X-chromosome inactivation) are characterized by a medium degree of methylation propensity or whether the underlying biological processes are so strong that basically every CpG island can become differentially methylated independently of its DNA sequence.

Besides these general observations, five more specific results are worth commenting on. First, in line with earlier observations we find almost all promoter CpG islands unmethylated, but also a significant number of intergenic CpG islands, which are often distant from any annotated gene. Little is known about the functional role of intergenic CpG islands. However, it has been observed that unmethylated CpG islands often co-localize with DNA replication origins [26], and we believe that it would be worthwhile to analyze the functional role of unmethylated intergenic CpG islands experimentally on a large sample. Methylation predictions may help to speed up and guide such an approach.

Second, we found evidence that the default methylation status of many CpG islands may be relatively stable during evolution. By comparing frequencies of the CGCG pattern to its (mutated) counterpart TGTG/CACA (the former is over-represented in unmethylated CpG islands of our dataset whereas the latter is over-represented in methylated CpG islands), we concluded that higher CpG →TpG mutation rates have applied to the CpG islands that we find methylated in human lymphocytes, than to those that we find unmethylated. Given that methylated CpG dinucleotides are more prone to CpG → TpG mutations [6], a straightforward explanation would be to postulate that the methylation status that we observe in our dataset (i) is similar to that found in the germline where mutations become fixed, and (ii) was stable over evolutionary time, so that the observed mutations could accumulate.

Third, our results show that certain aspects of DNA sequence and (predicted) DNA structure such as a high DNA rise and a low DNA twist seem to be associated with methylated CpG islands in vivo. It would be interesting to analyze how these sequence and structure attributes correlate with the in vitro recognition and methylation potential of CpG-rich sequences by mammalian DNA methyltransferases. Some reports suggest that unusual DNA structures (such as repeats and cruciform structures [27]) can lead to increased methylation activity by DNA methyltransferases. Moreover, local transitions between DNA in A-form, B-form, or Z-form may influence the methylation potential of the DNA, and it is tempting to speculate that some of our observed parameters may reflect such local differences in DNA structure formation.

Fourth, differences in error rates when training on the Chromosome 21 dataset and testing on the HEP dataset suggest that DNA-related characteristics identifying consistently methylated CpG islands are robust across tissues and genomic locations while those identifying unmethylated CpG islands are not—and have to be learned specifically for each tissue or genomic location. This interpretation is consistent with the hypothesis that most CpG islands in the human genome can become methylated, and do so if they are not preserved in the unmethylated state by specific (and tissue-dependent) influences, for example, the binding of transcription factors.

Fifth, we believe that our methodology for chromosome-wide correlation analysis and prediction is general enough to yield interesting results for other types of genomic and epigenomic data as well (such as histone modifications, replication origins, and many types of ChIP-on-Chip data). Therefore, we implemented our method as a web service which we will make accessible to interested researchers on a cooperation basis.

In conclusion, an understanding of the exact interplay between DNA-related features and CpG island methylation is likely to be of high practical and theoretical value. On the one hand, a reliable tool for predicting default CpG island methylation status from sequence would be of interest as a reference in cancer epigenetics and beyond. On the other hand, the fact that CpG island methylation is closely interwoven with certain features related to DNA sequence and structure—while minor changes such as SNPs seem to make little difference—may provide a key to uncovering the mechanisms that result in inter-individually similar and reproducible epigenetic reprogramming in the germline and the early embryo.

## Materials and Methods

### DNA methylation data.

This analysis is based on the results of a comprehensive measurement of CpG island methylation on human Chromosome 21 [10]. Briefly, Yamada et al*.* repeat-masked the chromosome sequence and computationally identified all non-repetitive CpG islands using standard tools and parameters (GC content above 50%, ratio of observed versus expected number of CpG dinucleotides above 0.6, more than 400 base pairs in length). Next, they designed primers for each identified CpG island and extracted corresponding DNA from samples of human peripheral blood lymphocytes. Finally, they determined the methylation status of each CpG island by methylation-specific restriction enzymes (via HpaII-McrBC-PCR). Yamada et al. validated their method by bisulphite sequencing of some CpG islands and concluded that it is highly reliable.

Their dataset comprises the methylation status of 149 CpG islands, each belonging to one of the following categories: fully methylated, unmethylated, incompletely methylated, or compositely/differentially methylated. Exploratory analysis using bisulphite sequencing indicated that the latter two classifications were not always unambiguous (unpublished data); therefore we focused on the two well-defined categories, fully methylated (31 cases) and unmethylated (103 cases). In order to minimize potential error sources, we re-mapped the boundaries of the CpGs islands that were originally used by Yamada et al*.* to the current human genome sequence (NCBI35) and we excluded two cases (both belonging to the fully methylated class) from the analysis because, in the light of this new mapping, the primers did not pick the intended CpG islands. Therefore, our dataset comprised 132 independent CpG islands, which are distributed relatively evenly across Chromosome 21 (see Dataset S2).

For validation, we also used data from the HEP pilot study [20]. In this study, Rakyan et al*.* determined the methylation status of 3,273 unique CpG dinucleotides (belonging to 253 amplicons) across seven tissues and one to eight samples per tissue by means of bisulphite direct sequencing. Out of these 253 amplicons, 210 could be mapped unambiguously to the NCBI35 genome version and had at least one measurement for each tissue. For these amplicons, we calculated average CpG dinucleotide methylation levels, both separately for individual tissue types and for all tissues combined. Those amplicons below a (arbitrarily chosen) threshold of 60% methylation were marked as unmethylated and those above this threshold were marked as methylated, resulting in a dataset of 163 “methylated” and 47 “unmethylated” amplicons.

### DNA-related attributes.

In order to identify DNA sequence-related attributes that are correlated with CpG island methylation, we compiled a comprehensive list of attributes that can be linked directly or indirectly to DNA sequence (the full list is given in Table S1). Most attributes take the form of frequencies or numerical scores, averaged over sequence windows and standardized to a default window size of one kb. They fall into eight biological classes, namely: (1) DNA sequence properties and patterns (428 attributes), (2) repeat frequency and distribution (494 attributes), (3) CpG island frequency and distribution (16 attributes), (4) predicted DNA structure (28 attributes), (5) gene and exon distribution (60 attributes), (6) predicted transcription factor binding sites (135 attributes), (7) evolutionary conservation (ten attributes), and (8) SNPs (13 attributes). The data for most of these attributes were collected from annotation tracks in the UCSC Genome Browser [28]. However, the attributes for class 1 were directly calculated from DNA sequence and the attributes for class 4 were calculated from DNA sequence by averaging over octamers [29] and trimers (J. Greenbaum, personal communication), respectively. We calculated these attributes for each CpG island in our dataset, both for the re-mapped CpG island itself and for 11 sequence windows around the CpG island: −20 kb to −10 kb, −10 kb to −5 kb, −5 kb to −2 kb, −2 kb to −1 kb, −1 kb to left boundary of CpG island, CpG island, right boundary of CpG island to +1 kb, +1 kb to +2 kb, +2 kb to +5 kb, +5 kb to +10 kb, +10 kb to +20 kb. Next, we removed those attributes that were zero in all cases (e.g., binding sites of rare transcription factors), giving us a list of 918 prediction attributes. To simplify the statistical analysis, we also removed attributes that were zero in most, but not all cases. For the CpG island level statistics (see next section), only the 706 attributes with non-zero values in the CpG island window of at least five methylated and five unmethylated cases were retained. For the sequence neighborhood statistics, only the 833 attributes were retained that had non-zero values in at least five methylated and five unmethylated cases, for at least four out of the 11 sequence windows.

### Statistics.

We performed statistical tests in order to determine attributes that exhibit significantly different values for fully methylated CpG islands compared to unmethylated CpG islands, at two levels. First, we compared all attributes at the CpG island level using the nonparametric Wilcoxon ranksum test (Dataset S1, first worksheet). Second, we compared all attributes across the complete sequence neighborhood of −20 kb to +20 kb around the CpG island (Dataset S1, second worksheet). To that end, quadratic regression functions were fitted over the attribute values in the 11 sequence windows around the CpG island (see previous section) and we used the ANOVA statistic to assess whether separate fitting for unmethylated versus methylated cases resulted in a significantly decreased error compared to combined fitting (quadratic regression functions were chosen to capture symmetry around the CpG island).

Significance thresholds were adjusted for multiple testing using the highly conservative Bonferroni method. Technically speaking, we controlled the family-wise error rate to be less than 1%.

### Prediction.

Machine learning methodology was used for two tasks: (i) to quantify the correlation between CpG island methylation and several classes of DNA-related attributes, and (ii) to predict CpG island methylation from the local genomic neighborhood.

The technical procedure is similar in both cases (cross-validation) and is discussed below. However, intention and interpretation differ for the two tasks. Task (ii) is the classical prediction scenario: given a dataset of limited size, we want to train a classifier for predicting CpG island methylation on unknown data and to quantify its expected prediction performance. Therefore, we train the classifier on the full set of 918 attributes, assuming that at least some of these attributes contain information that may be useful for the classifier.

In task (i), the goal is not so much to predict new data but to understand existing data. Here, we use a classifier as a tool to quantify the relationship between an attribute class (e.g., DNA sequence properties or repeats) and CpG island methylation. The rationale behind this is simple: If a classifier can successfully and reliably predict CpG island methylation using only information from one particular attribute class, then the attributes in this class are functionally associated with CpG island methylation and the prediction performance is a measure of the degree of functional association.

The prediction experiments follow essentially the same procedure. Given the list of CpG islands or amplicons and any selection of attributes from our list, a linear SVM is repeatedly trained to predict methylation status based on a 90% subset of cases, and its performance is evaluated on the remaining 10% of unseen cases. Technically speaking, we repeat 10-fold stratified cross-validation 20 times with different random partitions and sum the results on the test set (in terms of true- negatives, false-negatives, false-positives, and true-positives). The prediction performance is measured as the correlation coefficient between the predictions and the correct values on the test set. This criterion is commonly viewed as superior to comparing prediction accuracies because it is not as strongly affected by unbalanced class distributions [30].

For most prediction experiments (prediction setup A in Table 2), we used the linear SVM implementation provided by the WEKA package [31], which is based on the sequential minimal optimization method [32]. Additionally, several control experiments were performed that use different algorithms: an SVM with radial basis function kernel (from WEKA package, prediction setup B), AdaBoost M1 with decision tree stumps as the underlying classifier (from WEKA package, prediction setup C), the C4.5 tree generator (from WEKA package, prediction setup D), and a different implementation of a linear SVM (R implementation of LIBSVM [33], prediction setup E). All algorithms were applied with their suggested standard parameters.

### Experimental verification.

Predictions were performed using a linear SVM that was trained on the full Chromosome 21 dataset (132 cases) and all attribute classes. Subsequently, we determined the methylation status of 12 selected CpG islands by bisulphite sequencing as follows: initially, we applied direct sequencing of the PCR product to all 12 CpG islands (Table 3). In nine cases, this produced unambiguous results (i.e., very high conversion of CpGs = unmethylated, or almost no conversion = methylated). In the three remaining cases with mixed CG/TG sequencing profiles, PCR products were cloned and individual clones were sequenced in order to determine the methylation status. Average methylation was scored from single clone sequences using the BiQ Analyzer software [34]. Details of the experimental setting and the primers that we used are reported in Protocol S1. Human peripheral blood was obtained with the written consent of the donor.

## Supporting Information

Dataset S1 Attribute Statistics This Excel table reports raw *p*-values and multiple-testing-adjusted significance thresholds for all attribute statistics.
(388 KB XLS)

Dataset S2 DNA Methylation Data This Excel table contains a re-mapped and quality-controlled version of DNA methylation data that was originally reported by Yamada et al*.* [10], as it is used in this study. Furthermore, prediction accuracy and genome browser location are reported for all CpG islands.
(525 KB XLS)

Protocol S1 Experimental Validation This PDF document gives details on the experimental protocol that was used to determine the methylation status of the validation CpG islands, including PCR primers.
(26 KB DOC)

Table S1 Overview of Prediction Attributes This PDF document reports information on calculation, naming, and reference of all attributes that are used in this study.
Found at DOI: 10.1371/journal.pgen.0020026.st001 (89 KB DOC)

### Accession Numbers

The Ensembl database (http://www.ensembl.org/Homo_sapiens/index.html) accession numbers for the genes discussed in this paper are Ensembl gene *(ENSG00000197597)* and Ensembl gene *(ENSG00000160207).*

## Abbreviations

HEP: Human Epigenome Project
kb: kilobase
SNP: single nucleotide polymorphism
SVM: support vector machine
