# Supplementary material for: CpG Island Methylation in Human Lymphocytes Is Highly Correlated with DNA Sequence, Repeats, and Predicted DNA Structure
Source: PLoS Genet. 2006 Mar 3;2(3):e26. doi: 10.1371/journal.pgen.0020026 (PMC1386721; doi:10.1371/journal.pgen.0020026)
Supplement: Supplementary file 1 [file pgen.0020026.sd003.doc]

### Protocol S1 – Experimental validation

*Bisulphite treatment of genomic DNA*

500 ng *BamH*I digested genomic DNA was bisulphite-treated according to standard protocols [Frommer et al., 1992; Olek et al., 1996]. Briefly, denatured DNA was embedded into 2% agarose beads and treated with 2.7 M Na-metabisulphite for 3.5 h. After two washing steps with 1xTE desulphonation of the DNA was performed using 0.3 M NaOH followed by two subsequent washing steps with 1xTE. Bisulphite-DNA was directly used for amplification.

*PCRs*

Amplifications on bisulphate treated DNA were performed using the HotStarTaq DNA Polymerase from Qiagen in the presence of 1.5 mM MgCl_2_ according to the manufacturer´s protocol. Primers used for amplifications have the following sequences: C21orf99 5´ aag tta ggt agt ggg att tta gtt gt 3´, 5´ cca taa taa aaa cta caa aac taa acc caa 3´; ANKRD21 5´ ggt aag agt aat atg ggt att ttt gga gat 3´, 5´ ttt acc cca cca aac aac tct ata aaa 3´; STCH 5´ gaa tag gtt tgt gat gat tgt att 3´, 5´ aaa ata acc tat ttt ccc tca aat aaa 3´; CHODL 5´ ggt tta gtt ttt tat ttg tta gag gt 3´, 5´ cta act cta caa cct ccc ta 3´; CYYR1 5´ atg aaa tgt gag ttt ggg tgg tt 3´, 5´ acc att acc taa aaa acc ttc caa aa 3´; USP16 5´ ttg ttt ttt agg ggt tat ttt ggt 3´, 5´ aaa cta aca aaa cat aca acc aat aaa 3´; KRTAP6-2 5´ agg aag tat tta tat tga agt tat t 3´, 5´ ata tcc tca ata ata ata acc aaa a 3´; GART 5´ ttt gat tgg ttt taa agg gaa tgt t 3´, 5´ tat taa aac tcc caa aca cta cta a 3´; KCNJ6 5´ ttt agg gat gta gat ttt ttt ttt ttt 3´, 5´ aaa aaa atc ata att aaa taa aac cac 3´; CRYAA 5´ ttg ata gga gta gtt taa ttt aat agt t 3´, 5´ caa aaa tca cat acc tcc aac aaa 3´; PTTG1IP 5´ aga tga att ttt ttg ttt ttt gta ttg t 3´, 5´ ttc aaa aac aat taa aaa cta acc ca 3´; C21orf123 5´ ggt ttt ttt ttt ttt tga tgt gtt ttt 3´, 5´ ata cta ccc acc cat taa ctc aca aa 3´. The denaturation (95° C, 15 min) was followed by 40 PCR cycles (94° C 1 min, 1 min annealing [55° C, except of C21orf99 (58° C) and KRTAP6-2 (52° C)], 72° C 1 min), and a final extension at 72° C for 10 min.

PCR products were directly sequenced on a 3730x1 sequencer (ABI) at the *Max-Planck-Institut für Molekulare Genetik* in Berlin, Germany.

PCR products of the genes CHODL, CRYAA and PTTG1IP were cloned and sequenced on a CEQ8000 sequencer from Beckman-Coulter.

Frommer, M., L. E. McDonald, D. S. Millar, et al. (1992). "A genomic sequencing protocol that yields a positive display of 5-methylcytosine residues in individual DNA strands." Proc Natl Acad Sci U S A **89**(5): 1827-31.

Olek, A., J. Oswald and J. Walter (1996). "A modified and improved method for bisulphite based cytosine methylation analysis." Nucleic Acids Res **24**(24): 5064-6.
