# Supplementary material for: CpG Island Methylation in Human Lymphocytes Is Highly Correlated with DNA Sequence, Repeats, and Predicted DNA Structure
Source: PLoS Genet. 2006 Mar 3;2(3):e26. doi: 10.1371/journal.pgen.0020026 (PMC1386721; doi:10.1371/journal.pgen.0020026)
Supplement: Supplementary file 2 [file pgen.0020026.st001.doc]

### Table S1 – Overview of prediction attributes

0. *General remarks*

In order to make the attributes comparable between multiple CpG islands and windows of different sizes, most attributes are averages (or standard deviations, etc.) over the window of interest, standardized to a 1kb window. Variance stabilization and other more sophisticated methods are not performed.

Because many attributes share similar calculation patterns, we give an overview of typical calculations here, to which we will refer many times below.

*Distributions*:

_avg	=	mean

_std	=	standard deviation

_skew	=	skewness

_kurt	=	kurtosis

*Patches*:

Only feature patches are counted where the feature overlaps the window by at least 25% of the feature or 10% of the window. From the set of valid patches, the following attributes are calculated:

_len	=	total length of overlap with window, standardized to 1000 bps

_no	=	total number of patches, standardized to 1000 bps

_avg	=	average of full lengths, not only the overlap

_std	=	standard deviation of full lengths, not only the overlap

_sco	=	[only for *scored patches*] mean score (no weighting by length)

*Genes*:

Only genes are taken into account that overlap by at least one base pair with window. Attributes are then calculated at exon level for each exon that fulfils the patch overlap definition (overlap >25% of exon length or >10% of window size):

_elen	=	total length of exonic DNA with window, standardized to 1000 bps

_eno	=	total number of exons within window, standardized to 1000 bps

_eavg	=	average full length of the exons in the window

_estd	=	standard deviation of full lengths of the exons in the window

(no weighting by genes)

All genes that contain at least one exon fulfilling the above condition are taken into account for the gene statistics.

_glen	=	total length of exonic+intronic DNA with window, standardized to 1000 bps

_gno	=	total number of genes within window, standardized to 1000 bps

_gavg	=	average full length of the genes in the window

_gstd	=	standard deviation of full lengths of the genes in the window

_gcav	=	average number of exons per gene

_gcsd	=	standard deviation of exons per gene

1. *DNA sequence properties and patterns*

| **Attribute Name(s)** | **Description** | **Implementation / Reference** |
| --- | --- | --- |
| Pat_AAAA to  Pat_TTTT | Pattern frequency (strand-specific, +-strand only) of all 4-mers | By simple counting on the +-strand of the genomic sequence (NCBI35) |
| P_TATATA etc. | Pattern frequency (strand-specific, +-strand only) of a few artificially picked special purpose patterns | By simple counting on the +-strand of the genomic sequence (NCBI35) |
| Uni_AAAA  To Uni_CTTT | Pattern frequency (not strand-specific, both strands) of all 4-mers | By simple counting on the +-strand and saving to either the pattern or the reverse complement, whichever comes earlier in the alphabet |
| fG_avg  fG_std  fG_skew  fG_kurt fC_avg  fC_std  fC_skew  fC_kurt  fCG_avg  fCG_std  fCG_skew  fCG_kurt  fOE_avg  fOE_std  fOE_skew  fOE_kurt | Frequency distribution of Cs, Gs, CpGs, and of the Observed / Expected ratio | Calculate directly from sequence (NCBI35), over non-overlapping 50bp windows and calculate average, std-dev, skewness, and kurtosis for the resulting distribution |

2. *Repeat frequency and distribution*

| **Attribute Name(s)** | **Description** | **Implementation / Reference** |
| --- | --- | --- |
| WDu_len  WDu_no  WDu_avg  WDu_std | Confirmed segmental duplications, defined as having similarity to sequences in the Segmental Duplication Database (SDD) | From UCSC Genome Browser “WSSD Duplication Track”: celeraDupPositive.ChromStart and ChromEnd in the usual way for patches |
| SDu_len  SDu_no  SDu_avg  SDu_std  SDu_sco | Duplications of at least 1 Kb of the total sequence (containing at least 500 bp of non-RepeatMasked sequence) with a sequence identity of at least 90% | From UCSC Genome Browser “Segmental Duplications”: genomicSuperDups.ChromStart and ChromEnd in the usual way for scored patches |
| SAl_len  SAl_no  SAl_avg  SAl_std  SAl_sco | Alignments of the human genome with itself, using a gap scoring system that allows longer gaps than traditional affine gap scoring systems | From UCSC Genome Browser “Self Chain”: chr21_chainSelf.ChromStart and ChromEnd in the usual way for scored patches |
| Rep_len  Rep_no  Rep_avg  Rep_std  Rep_sco  Rep_ssco  Rep_sta  Rep_ssta  Rep_end  Rep_send  Rep_lef  Rep_slef  RCl_XXXX  RCn_XXXX  RCa_XXXX  RCs_XXXX  RC1_XXXX  RC2_XXXX  RC3_XXXX  RC4_XXXX  RC5_XXXX  RC6_XXXX  RC7_XXXX  RC8_XXXX  RFl_XXXX  RFn_XXXX  RFa_XXXX  RFs_XXXX  RF1_XXXX  RF3_XXXX  RF5_XXXX  RF7_XXXX | Repeats found by RepeatMasker, separate for all repeats, repeats by class (SINE, LINE, etc.), and repeats by family (L1, L2, Alu, etc.)  Rep_sco = mean of Smith-Waterman alignment score (1)  Rep_ssco = std. dev. (2)  Rep_sta = alignment start relative to full repeat (3)  Rep_ssta = std. dev. (4)  Rep_end = alignment end relative to full repeat (5)  Rep_send = std. dev. (6)  Rep_lef = no. of bases of full repeat that are unaligned (7)  Rep_slef = std. dev. (8)  (for classes: mean and stddev; for families: only mean) | From UCSC Genome Browser “RepeatMasker”: chr21_rmsk in the usual way for scored patches |
| Tan_len  Tan_no  Tan_avg  Tan_std  Tan_per  Tan_sper  Tan_cop  Tan_scop  Tan_sco  Tan_ssco  Tan_ent  Tan_sent | Simple tandem repeats (possibly imperfect) located by Tandem Repeats Finder  Period, mean + std. dev  Copy number, mean + std. dev  Score, mean + std. dev  Entropy, mean + std. dev | From UCSC Genome Browser “Simple Repeats”: simpleRepeat in the usual way for scored patches |

3. *CpG island frequency and distribution*

| **Attribute Name(s)** | **Description** | **Implementation / Reference** |
| --- | --- | --- |
| CpG_len  CpG_no  CpG_avg  CpG_std  CpG_gc  CpG_sgc  CpG_rat  CpG_srat | Prevalence and characteristics of CpG islands in the vicinity of the CpG island under consideration  CpG island parameters:  GC > 50%, length > 400 bp (!), Obs/Exp > 0.6, no repeat masking  Explanation:  CpG_gc = *avg. CpG percentage*  CpG_rat = *avg. CpG observed / expected ratio* | From UCSC Genome Browser “CpG Islands”: CpGIslandExt in the usual way for scored patches (after manual removal of CpG islands of less than 400 bp) |
| mCG_len  mCG_no  mCG_avg  mCG_std  mCG_gc  mCG_sgc  mCG_rat  mCG_srat | Same as above but with a modified CpG island definition: GC > 55%, length > 200 bp (!), Obs/Exp > 0.6, no repeat masking | Calculated with the help of a locally modified version of “CpG_searcher.pl” by Daiya Takai and Peter A. Jones |

4. *Predicted DNA structure properties*

| **Attribute Name(s)** | **Description** | **Implementation / Reference** |
| --- | --- | --- |
| SASA_avg  SASA _std  SASA _skew  SASA _kurt | Solvent accessible surface area of the DNA | Predict for each base from the DNA sequence according to “Greenbaum (BU) solvent accessibility prediction”, based on “22_Solvent_ Accessibility_Trimers.txt”. Calculate average, std-dev, skewness, and kurtosis of the 1 bp resolution distribution in window |
| twis_avg  twis_std  twis_skew  twis_kurt  roll_avg  roll_std  roll_skew  roll_kurt  tilt_avg  tilt_std  tilt_skew  tilt_kurt  rise_avg  rise_std  rise_skew  rise_kurt  slid_avg  slid_std  slid_skew  slid_kurt  shif_avg  shif_std  shif_skew  shif_kurt | Basic structural properties of the DNA | Predict from the DNA sequence based on “23_Octamer_Structure_ Attributes.txt” over non-overlapping 20bp windows and calulate average, std-dev, skewness, and kurtosis for the resulting distribution |

5. *Gene and exon distribution*

| **Attribute Name(s)** | **Description** | **Implementation / Reference** |
| --- | --- | --- |
| Reco_avg  Reco_std | Average recombination rate over a 1mb window | From UCSC Genome Browser “Chromosome Band Track” (deCODE genetic map): recombRate.decodeAvg, taking the unweighed average and stddev of overlapping areas |
| CCD_elen  CCD_eno  CCD_eavg  CCD_estd  CCD_glen  CCD_gno  CCD_gavg  CCD_gstd  CCD_gcav  CCD_gcsd | Human genome high-confidence gene annotations from the Consensus CDS (CCDS) project | From UCSC Genome Browser “CCDS”: ccdsGene in the usual way for genes |
| Ref_elen  Ref_eno  Ref_eavg  Ref_estd  Ref_glen  Ref_gno  Ref_gavg  Ref_gstd  Ref_gcav  Ref_gcsd | Known protein-coding genes taken from the NCBI mRNA reference sequences collection (RefSeq) | From UCSC Genome Browser “RefSeq Genes”: refFlat in the usual way for genes |
| Ens_elen  Ens_eno  Ens_eavg  Ens_estd  Ens_glen  Ens_gno  Ens_gavg  Ens_gstd  Ens_gcav  Ens_gcsd | Ensembl gene annotations | From UCSC Genome Browser “Ensembl Genes”: ensGene in the usual way for genes |
| Yale_len  Yale_no  Yale_avg  Yale_std | Yale Pseudogene annotation | From UCSC Genome Browser “Yale Pseudo”: pseudoYale in the usual way for patches |
| Retr_len  Retr_no  Retr_avg  Retr_std  Retr_sco | UCSC retrotransposed gene annotation | From UCSC Genome Browser “Retrotransposed Genes”: pseudoGeneLink in the usual way for scored patches |
| ExA_len  ExA_no  ExA_avg  ExA_std  ExA_sco  ExA_ssco  ExA_var  ExX_sco  ExX_ssco | Degree of expression (the ratio over the median of several replicates) according to the GNF Atlas 2, averaged over all tissues (A) resp. for interesting groups of cells (X according to “categ” column in “12_GNF_Atlas _2_tissue_manual”) | From UCSC Genome Browser “GNF Atlas 2 Track”: gnfAtlas2 gives the values (column expScores) and hgFixed.gnfHumanAtlas2MedianExps gives the tissue information necessary for aggregation, in the way that the first value in expScore corresponds to tissue id 0, second to tissue id 1, … until 79)  The attributes are calculated in an adapted way for scored patches:  Explanation:  ExA_sco = *avg. of expression of probed sequences in window*  ExA_sstd = *stddev of ExA_sco over probed sequences in window (no weighting)*  ExA_var = *avg. of (std. dev. over tissues) over genes in window* |

6. *Predicted transcription factor binding sites:*

| **Attribute Name(s)** | **Description** | **Implementation / Reference** |
| --- | --- | --- |
| TF_Total  TF_XXX | Number of human/mouse/rat conserved, computationally identified, transcription factor binding sites in window (XXX stands for any transcription factor with a known binding matrix – all TFs starting with the same three letters are assumed to for a group and are counted together) | From UCSC Genome Browser “TFBS Conserved”: tfbsConsSites by simple counting (after manual removal of TFBS that have a frequency of less than 5 on chrom21q). To get some aggregation, TF where the first three letters are identical are put into the same category |

7. *Evolutionary conservation*

| **Attribute Name(s)** | **Description** | **Implementation / Reference** |
| --- | --- | --- |
| Mos_len  Mos_no  Mos_avg  Mos_std  Mos_sco  Mos_ssco | Elements of high conservation as calculated by PhastCons over pairwise Human/Chimp/Mouse/ Rat/Dog/Chick/Fugu/Zfish  whole genome alignments | From UCSC Genome Browser “Most Conserved”: phastConsElements in the usual way for scored patches |
| Con_avg  Con_std  Con_skew  Con_kurt | Conservation score distribution | From UCSC Genome Browser “Conservation Track”:  Calculate average, std-dev, skewness, and kurtosis of the 1 bp resolution distribution in window |

8. *Single nucleotide polymorphisms*

| **Attribute Name(s)** | **Description** | **Implementation / Reference** |
| --- | --- | --- |
| S_count  SC_XXXXX  SF_YYYYY | Total number of SNPs in window, number of SNP by class (snp, in-del, …), and by function (manually simplified) | From UCSC Genome Browser “SNPs”: snp by simple counting and use of the first 5 characters of ‘class’ as XXXXX and of ‘func’ as YYYYY |
